# WHEN DO FRIENDS MATTER? MODERATORS OF THE ASSOCIATION BETWEEN SOCIAL SUPPORT FROM FRIENDS AND DEPRESSIVE SYMPTOMS

**DOI:** 10.1093/geroni/igae098.1529

**Published:** 2024-12-31

**Authors:** Xinfang Yu, Shannon Ang

**Affiliations:** Nanyang Technological University, Singapore, Singapore; Nanyang Technological University, Singapore, Singapore

## Abstract

With shrinking family sizes in many parts of the world, older adults are beginning to rely more on friendships for support in later life. Because individuals get to choose their own friends (unlike family), the typical assumption is that perceiving more social support from friends is protective of mental health. In this study, we examine if the association between social support from friends and depressive symptoms is contingent on other factors, such as age and support from family. We use three waves of data (2014-2018) from a nationally representative study of older adults aged 60 years and above in China (N = 6,304 person-year observations). We estimated growth curve models with an accelerated longitudinal design, using interaction terms to assess whether age, friends, and family ties moderate the relationship between support from friends and depressive symptoms. The results suggest that the protective effects of friend support on depressive symptoms reduces with increasing age and greater family support. We also found the relationship between social support from friends and depressive symptoms was curvilinear – the direction of the relationship depended on the degree of support perceived. Our findings provide a more nuanced view of the benefits of friendships for psychological well-being, suggesting moderation in the positive effects of friend support despite the common assumption that they are always beneficial. Understanding how social support from friends contributes to their health can help guide efforts to maximize gains and minimize losses that matter most to people as they age.

